# Strong, Ductile, and Thermally Stable bcc-Mg Nanolaminates

**DOI:** 10.1038/s41598-017-08302-5

**Published:** 2017-08-15

**Authors:** Siddhartha Pathak, Nenad Velisavljevic, J. Kevin Baldwin, Manish Jain, Shijian Zheng, Nathan A. Mara, Irene J. Beyerlein

**Affiliations:** 1 Chemical and Materials Engineering, University of Nevada, Reno, NV USA; 2 Shock and Detonation Physics, Los Alamos National Laboratory, Los Alamos, NM USA; 3 Center for Integrated Nanotechnologies, Los Alamos National Laboratory, Los Alamos, NM USA; 4 Shenyang National Laboratory for Materials Science, Institute of Metal Research, Chinese Academy of Sciences, Shenyang, 110016 China; 5 Institute for Materials Science, Los Alamos National Laboratory, Los Alamos, NM USA; 6 Mechanical Engineering Department, Materials Department, University of California at Santa Barbara, Santa Barbara, CA 93106 USA

## Abstract

Magnesium has attracted attention worldwide because it is the lightest structural metal. However, a high strength-to-weight ratio remains its only attribute, since an intrinsic lack of strength, ductility and low melting temperature severely restricts practical applications of Mg. Through interface strains, the crystal structure of Mg can be transformed and stabilized from a simple hexagonal (hexagonal close packed hcp) to body center cubic (bcc) crystal structure at ambient pressures. We demonstrate that when introduced into a nanocomposite bcc Mg is far more ductile, 50% stronger, and retains its strength after extended exposure to 200 C, which is 0.5 times its homologous temperature. These findings reveal an alternative solution to obtaining lightweight metals critically needed for future energy efficiency and fuel savings.

## Introduction

Magnesium (Mg) is the lightest weight structural metal, and being 35% lighter than Al and 78% lighter than steel, it has tremendous potential for achieving higher energy efficiency, particularly in the aerospace and automotive industries^1, 2^. However unlike steel, Mg is inherently not ductile and formable, and thus cannot be shaped into parts for structural components^3, 4^. This intrinsic limitation is linked to its low symmetry hexagonal close packed (hcp) crystal structure (‹c›/‹a› = 1.623). Plastic slip in hcp metals must occur on atomic planes and directions with significantly different activation barriers^5, 6^. Critical stresses to activate the few available slip systems in the ‹a› direction are several times smaller than those to activate the 12 slip systems in the ‹c + a› direction^7–9^. This severe anisotropy is the fundamental cause for the low ductility and formability of Mg. This is in stark contrast to the more familiar and more formable steel, which has a symmetric body center cubic (bcc) crystal structure and at least 48 available slip systems with similar activation stresses^10^.

To make hcp Mg more formable, intense research over the years has been dedicated to exploring whether the critical stress differences among the hcp slip systems can be reduced via alloying^11–13^ or nanostructuring^4^. Yet still the hcp crystal structure renders the atomic cores of the dislocations difficult to move through the perfect lattice^3^. Here we consider a different, more radical approach to overcoming the strength and ductility problem. Interface strain engineering of Mg with Nb is exploited to transform hcp Mg into stable bcc Mg at ambient pressures and the adjacent Mg/Nb interfaces are spaced within a few nanometers forming multilayered Mg/Nb nanocomposite. It is postulated that the bcc structure of the Mg phase enables significant increases in its ductility from that of conventional hcp Mg and the nanostructure will bring about an order of magnitude increase in strength, while retaining the lightweightness (high strength-to-weight ratio) of elemental Mg.

As reported in this letter, we have carried out experiments to investigate the strength, deformation behavior, and thermal stability of bcc Mg within a nanolayered composite. Magnetron sputtering is used to synthesize 5 nm/5 nm Mg/Nb nanocomposites. We demonstrate that the Mg present in the 5 nm/5 nm Mg/Nb nanocomposite is entirely bcc, without any trace of hcp Mg. This is different from other prior works^14^ where interface strains were successful in transforming only portions of Mg to the bcc structure. Our present work is unique in that it allows us to distinguish cleanly between the mechanical behavior of the transformed bcc phase from the untransformed hcp Mg. We show via nanomechanical testing, that bcc nano-Mg leads to a 40% higher strength and 125% increase in strain to failure as compared to 50 nm/50 nm Mg/Nb nanocomposites (where Mg has an hcp structure) made by the same method. Thermal stability tests demonstrate further that despite the finer layer size, the bcc Mg/Nb nanocomposite has exceptionally high thermal stability (at 0.5 times the homologous temperature). These results reveal an exciting potential for a new bcc phase of Mg to be lightweight, strong, ductile, and thermally stable, overcoming many of the bottlenecks with conventional hcp Mg.

Use of interface strains is the only technique through which pure Mg can be made to exist in the bcc phase at ambient temperature and pressures. Although alloying is known to stabilize Mg in cubic (both bcc and face centered cubic, fcc) structures^11–13^, pure Mg can exist in the bcc structure only under enormously high pressures (50 ± 6 GPa)^15, 16^. It has been shown previously that bcc Mg can develop locally in biphase nanolayered films made by deposition provided that the individual layer thicknesses becomes sufficiently fine, around 5 nm^14^. A balance of strain energy in the Mg and the energy of the interface formed with the neighboring dissimilar material drives the phase transformation, and hence the bcc Mg in the nanolayer is intrinsically different from the bcc (bulk) Mg that forms as a high-pressure phase^17^. The interface strain energy approach has been also used to stabilize unconventional crystal structures phases within other very fine nanolayered two-phase composites. For instance, bcc Zr in Zr/Nb nanolayered films have been reported when 2 *h* < 3.1 nm (where *h* is the individual layer thickness)^18, 19^, fcc Nb in Cu/Nb layers^20^, and fcc Ti in Ti/Al and Ti/Ag composites^21–23^.

**Figure 1:**
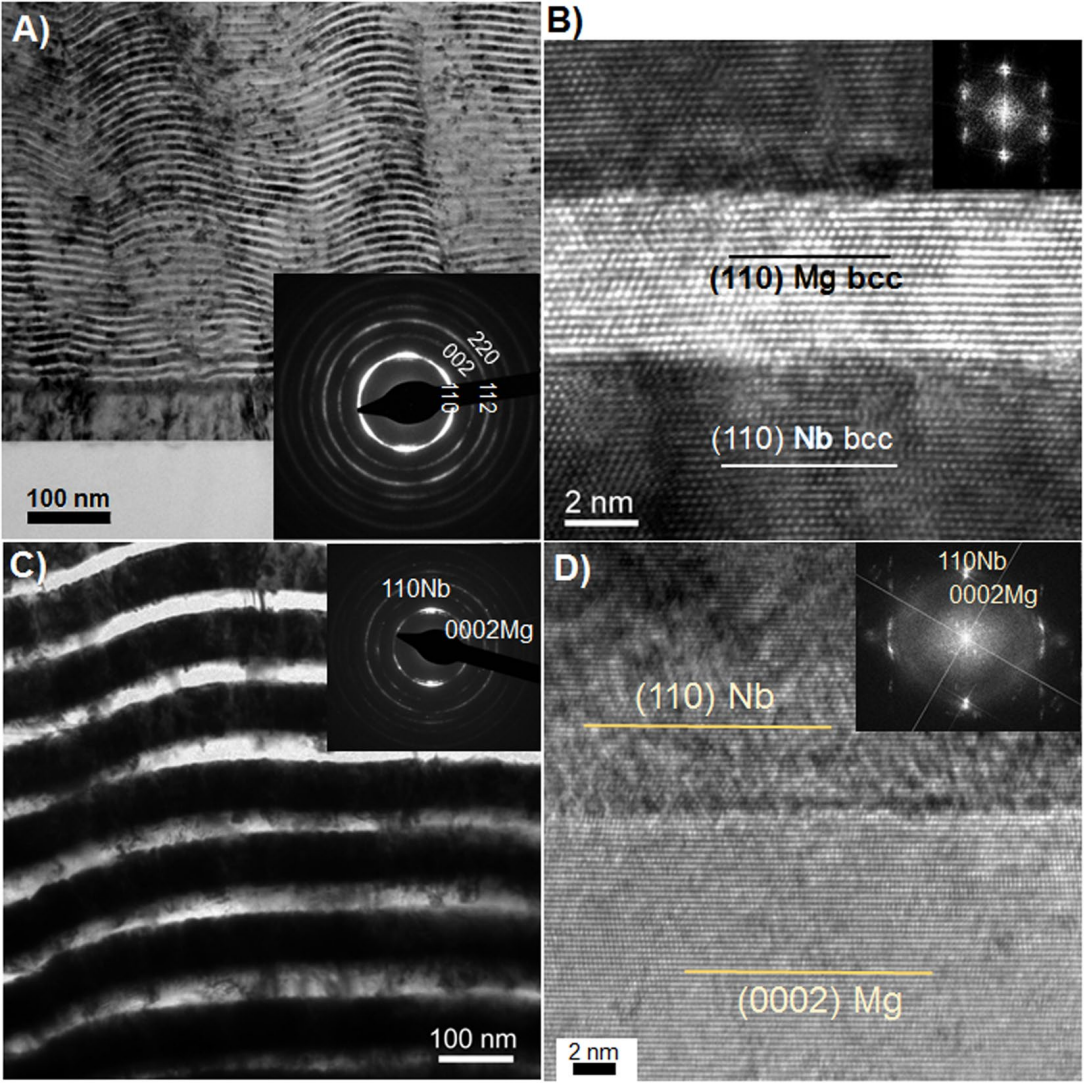
(**a**,**b**) TEM of the 5 nm/5 nm bcc/bcc and (**c**,**d**) the 50 nm/50 nm hcp/bcc Mg/Nb nanocomposite.

Thin multilayers composed of alternating Mg and Nb layers were deposited at room temperature using direct current (dc) magnetron sputtering on Si substrates with the equal (targeted) volume fraction of Mg:Nb = 1:1. Nb was chosen due to its inherent bcc crystal structure under ambient conditions with a lattice parameter (‹a› 3.3 Å) smaller than that of (bulk) bcc Mg (‹a› 3.571 Å), and since it is immiscible with Mg even at elevated temperatures, and its higher stiffness, strength and hardness as compared to Mg. These have been suggested as necessary criteria for stabilization of bcc Mg in a biphase nanolaminate architecture^17^. In order to compare bcc-Mg with hcp-Mg, multilayers were deposited for two different individual (targeted) layer thicknesses: 5 nm/5 nm and 50 nm/50 nm Mg/Nb nanocomposites. Transmission electron microscopy (TEM) was used to measure the actual layer thicknesses (Fig. 1). The 5 nm/5 nm Mg/Nb nanocomposite was found to have almost equal Mg and Nb layer thicknesses of about 5.5 nm (Fig. 1a and b), and the 50 nm/50 nm Mg/Nb nanocomposite had a Mg layer thickness of ~35 nm and a Nb layer thickness ~65 nm (Fig. 1c and d). For the 5 nm/5 nm composite, the layers appear to undulate (Fig. 1a); however, the wavelength is approximately 150–200 nm, much greater than the individual layer thicknesses.

These two choices for layer thickness were based on prior analytical and DFT calculations that suggested that the bcc Mg phase can be maintained up to a critical value of 4.2 nm from the interface^24^. For a nanolayered composite, this calculation corresponds to a critical Mg layer thickness of ~8.4 nm, and thus layer thicknesses of 5 nm and 35 nm for Mg are well below and above this critical value.

**Figure 2:**
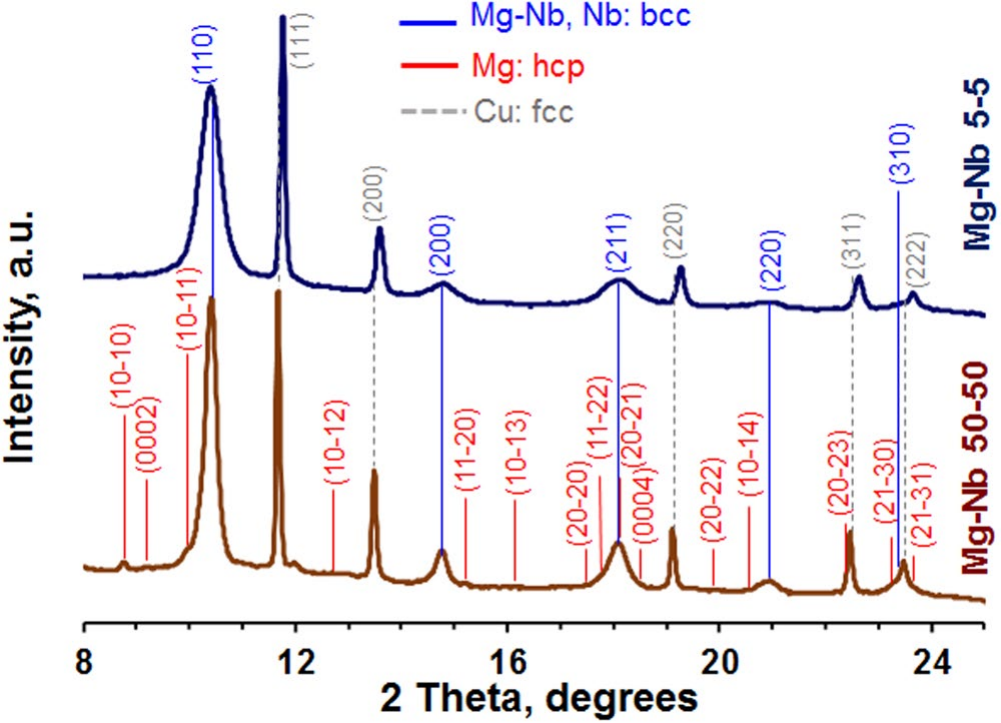
XRD of the 5 nm/5 nm bcc/bcc and the 50 nm/50 nm hcp/bcc Mg/Nb nanocomposite. Copper was used as a pressure marker during these experiments.

Multiple angle dispersive x-ray diffraction measurements were made in order to resolve the crystal structure composition of the sample (Fig. 2). All x-ray diffraction measurements were performed using the synchrotron x-ray beam at the Advanced Photon Source at Argonne National Laboratory. A monochromatic x-ray beam with a 5 μm by 5 μm FWHM and 0.4246 Å wavelength, and a MAR345 image plate area detector were used in all measurements. To ensure that adequate sampling volume and crystal orientations were measured, for each x-ray spectra, the sample was continuously rotated +/−30° and with a 50 μm × 50 μm grid raster with respect to the input x-ray beam. The diffraction results on the 50 nm/50 nm material indicate that the Mg layers in this composite have a hcp crystal structure. Most importantly, they show that in the 5 nm/5 nm composite the Mg is uniformly bcc, wherein no hcp peaks were detected. The X-ray synchrotron (XRS) measurements also provide the lattice parameter for both phases. In the 50/50 nm composite, we find that the lattice parameters of Mg and Nb in the nanocomposite (‹a› = 3.228 Å and ‹c› = 5.306 Å for Mg (‹c›/‹a› = 1.644) and ‹a› 3.318 Å for Nb) are similar to that of bulk (non-laminated) Mg and Nb (‹a› = 3.21 Å and ‹c› = 5.21 Å for bulk Mg (‹c›/‹a› = 1.623^25^) and ‹a› 3.304 Å for bulk Nb^26^). In the 5/5 nm composite, both phases adopted the same ‹a› = 3.347 Å. This value is similar to that of bulk Nb (3.304 Å^26^), but deviates considerably from that of the high-pressure bcc Mg phase (2.953 Å^15^, or 2.78–2.84 Å^16^). These measurements indicate that the bcc Mg phase formed in the nanolayer is fundamentally different from the bulk high-pressure bcc Mg phase reported in literature^17^, and hence deserves further study. Next, we proceed to study the unique properties of this hitherto-unstudied bcc Mg phase.

From the diffraction patterns in Fig. 2, we can obtain information on the Mg/Nb interface crystallographic character that occurs in each composite. First, we observe from the measurement that both phases in both composites are highly textured, which suggest the possibility that the 50 nm/50 nm composite possessed a {0001}Mg||{110}Nb predominant interface and the 5 nm/5 nm had a prevailing {110}Mg||{110}Nb interface. High resolution HR (HR-TEM) was used to confirm these findings (Fig. 1b and d). Consistent with the inference from the strongly preferred XRS texture, the interfaces in the 50 nm/50 nm composite are {0001}Mg||{110}Nb (Fig. 1d). For the 5/5 nm composite, the HR TEM analysis confirms that the crystal structure of Mg in the 5 nm is bcc. For this material, the XRS measurements found an equivalent ‹a› between Mg and Nb suggesting that the Mg/Nb interface is coherent. The HR-TEM analysis finds that the Mg/Nb interface is {110}Mg||{110}Nb with a cube-on-cube orientation relationship (Fig. 1b).

Creating bcc Mg requires understanding a few key processing parameters that can affect the critical Mg layer thickness for the interface-induced hcp→bcc transition. Here we have identified two: (a) the underlying substrate on which the thin films are deposited, and (b) intrinsic film stresses after deposition. A series of depositions were carried out on various substrates, such MgO (100), MgO (111), sapphire A plane, sapphire C plane and Si (100), in order to check the texture of the deposited films. Substrates that showed traces of Mg/Nb interface with orientations other than {110}Mg||{110}Nb were ignored in subsequent analysis. The resultant intrinsic film stresses were also calculated for each film-substrate condition using wafer curvature profilometery measurements^27, 28^, and the measurements were repeated for every change in deposition conditions such as changes in deposition temperature, pressure, bias and deposition time (film thickness). The goal here was to maintain the intrinsic film stress levels to as close a stress-free condition as possible, since defects can appear when the intrinsic stresses exceed the strength of the film-substrate interface^29^. Under extreme cases, such defects manifest themselves as buckles in the film in the case of a compressive stress, and cracks if tensile stresses are present. But even moderate level of these stresses can lead to an incomplete hcp→bcc transition^14^. For the purpose of this study the Si (100) substrate was chosen as it provided the best combination of a {110}Mg||{110}Nb for the 5 nm/5 nm Mg/Nb nanocomposite, without any trace of hcp Mg, and a relatively low intrinsic film stress of around 200 MPa (compressive).

**Figure 3:**
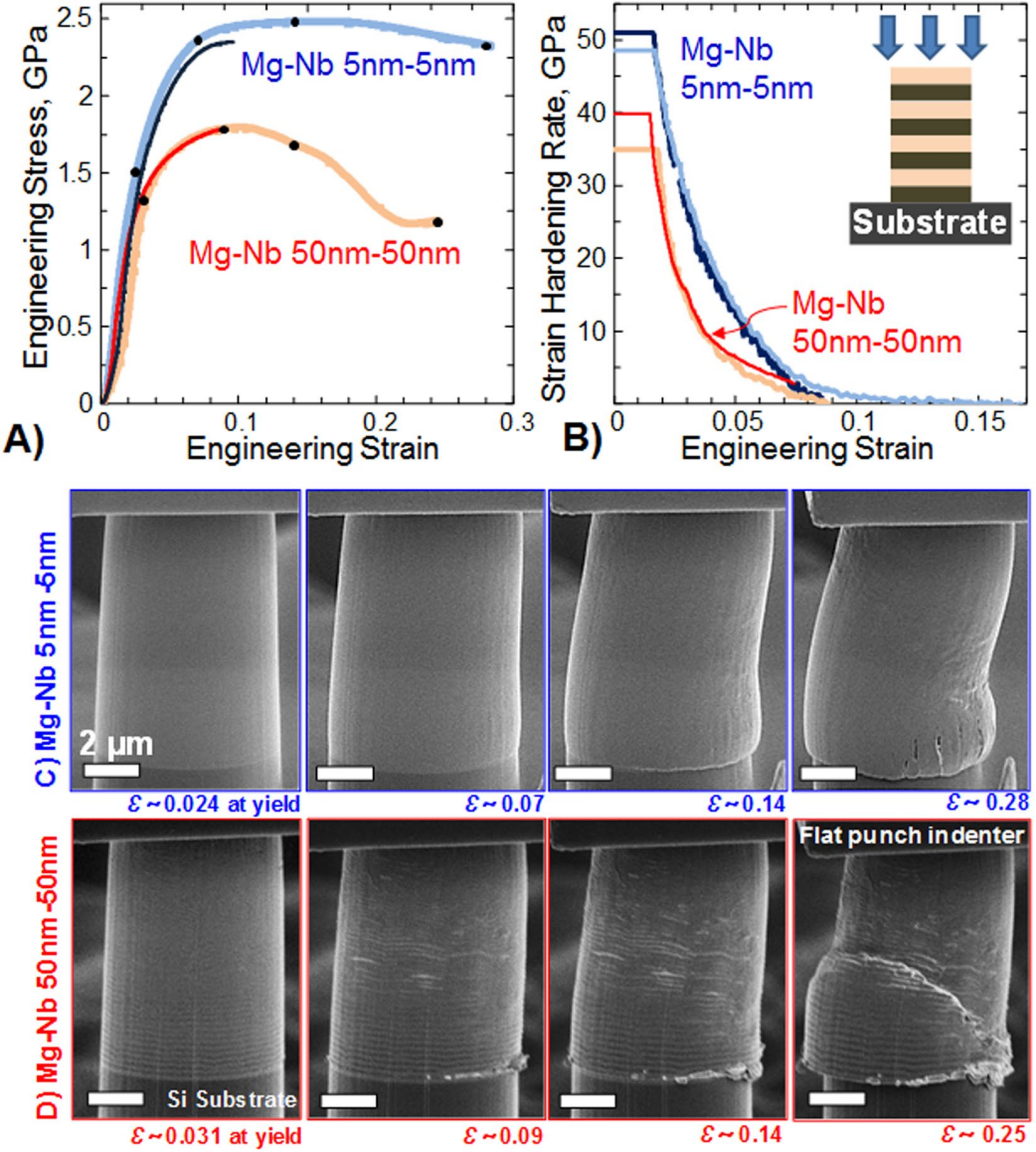
(**a**) Engineering stress-strain curves and (**b**) strain hardening rates obtained from micropillar compression tests of 5 nm/5 nm bcc/bcc and 50 nm/50 nm hcp/bcc Mg/Nb nanocomposites. Two representative tests for each layer thickness, one stopped at lower strains (*ɛ* ~ 0.1) and another at higher strain levels (*ɛ* ~ 0.25–0.3), are shown in order to demonstrate the repeatability of the results. (**c** and **d**) The micropillar compression process was recorded as a video file from which individual picture frames were extracted corresponding to strain levels of (**c**) *ε* ≈ 0.024, 0.07, 0.14 and 0.28 for the 5 nm/5 nm Mg/Nb nanocomposite and (**d**) *ε* ≈ 0.031, 0.09, 0.14 and 0.25 for the 50 nm/50 nm Mg/Nb nanocomposite (as indicated by the black dots on the stress-strain graph in (**a**)).

To compare the deformation behavior of bcc nanoMg with hcp nanoMg, micropillars were fabricated from both the 5 nm/5 nm and 50 nm/50 nm nanocomposites using focused-ion beam (FIB) micromachining and tested in compression normal to the Mg/Nb interface planes (Fig. 3). The elastic deformation of the Si (100) substrate (i.e., the machine compliance) was measured from the recorded images. Subtracting the machine compliance from the stress-strain data allows us to accurately calculate the composite moduli from these tests. It is typically difficult to locate the point of full contact during loading between the indenter and the micropillar (due to misalignment of the indenter tip with respect to the pillar). Hence only the linear unloading portions of the stress-strain data were used for modulus calculations. Only tests that were unloaded at low strain levels (*ϵ* ~ 0.1) were used in the modulus calculations.

Figure 3a shows the engineering stress-strain curves obtained from the micropillar tests on both nanocomposites. Two representative stress-strain curves for each layer thickness are shown in Fig. 3a and b, demonstrating the repeatability of the results. For the 5 nm/5 nm nanocomposite, the average modulus and 0.2% offset yield stress values were measured to be *E* = 75.11 ± 3.4 GPa (average ± standard deviation) and *Yield stress* = 1.41 ± 0.1 GPa, and for the 50 nm/50 nm nanocomposite, the values were *E* = 69.48 ± 3.3 GPa and *Yield stress* = 1.24 ± 0.9 GPa respectively. The measured modulus values for the 50 nm/50 nm nanocomposite (where Mg has an hcp structure) match well with the expected isostress modulus of 71.30 GPa, for this composite calculated from composite theory^30^.

**Figure 4:**
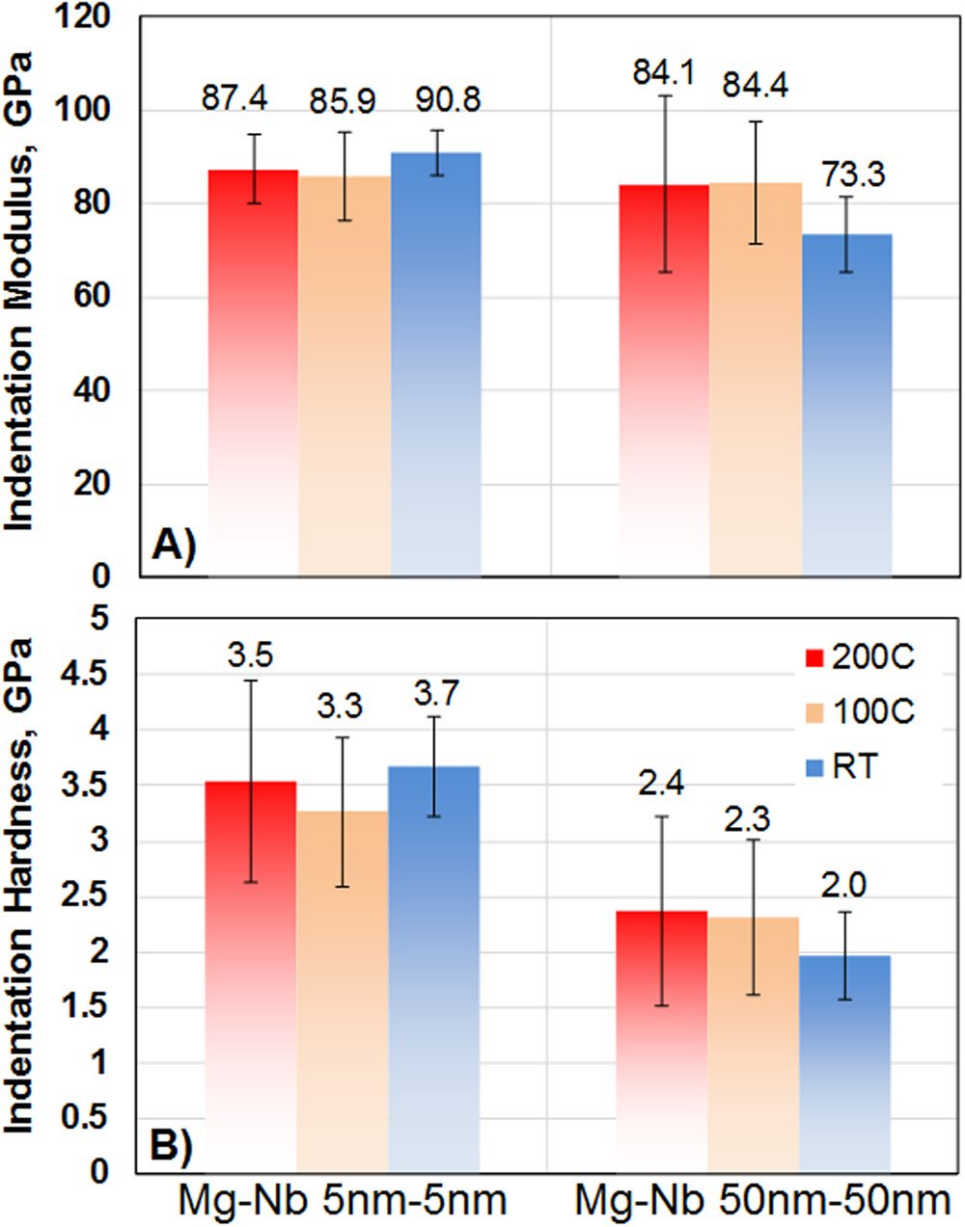
(**a**) Indentation modulus and (**b**) hardness (average ± standard deviation) of 5 nm/5 nm and 50 nm/50 nm Mg/Nb nanocomposites before and after exposure to high-temperatures of 100 °C and 200 °C.

The above numbers indicate that both nanocomposites are much stronger (at least 10 times) than that of coarse-grained Mg or Nb or a volume average of their strengths^31, 32^. While noteworthy, this is an expected trend for nanostructured metals (“smaller is stronger”). As an appropriate comparison, we utilize the yield and hardness values for nanocrystalline Mg (hardness 0.5 GPa for a 44 nm grain size^33^ and yield strength 0.16 GPa for 42 ± 5 nm grain size^34^) and nanocrystalline Nb (hardness 2.53 GPa for grain size range of 25–220 nm^35^). Remarkably, the values shown in Figs 3 and 4 are 2–3 times higher than the volume average of the nanocrystalline counterparts (yield stress 0.5–0.6 GPa and hardness 1.5–1.8 GPa), suggesting different strengthening mechanisms in an interface dominated architecture^36^. The exceptionally high yield strengths and hardness values (3.7 ± 0.5 GPa, Fig. 4) for the lower thickness 5 nm/5 nm deserve special mention; these values are 47% higher than those reported in literature for similar layer thicknesses but bearing an incomplete hcp→bcc transition of Mg^14^.

We expect the deformation mechanisms between the Mg/Nb 5 nm/5 nm and 50 nm/50 nm nanolaminates to vary significantly due to three primary reasons: (i) the change in crystal structure from hcp to bcc, (ii) due to the reduction in layer thickness from 50 nm to 5 nm, a size effect, or specifically the ‘smaller is stronger’ effect that reduces the number of dislocations that can pile up at the interfaces, and (iii) differences in interface behavior to the imposed deformation, particular in response to its interaction with dislocations.

The compelling finding here lies in the nearly 14% increase in the yield stress values and an almost 43% increase in peak strength when going from the hcp Mg to the bcc Mg composite. The peak strength is defined as the maximum stress value beyond which instability is observed during the micropillar compression; the corresponding strain value is designated as the strain to failure (or strain to instability). Beyond this point, the stress state in the micropillar deviates significantly from uniaxial compression. Some of this strengthening is likely a consequence of the reduction in layer thickness from 50 nm to 5 nm alone. In many other bimetal nanocomposite systems strengthening often accompanies decreases in layer thickness *h*, particularly in the range of 100 nm > *h* > 5 nm^36^. However, among hcp Mg crystals of nanoscale dimensions, the reported grain size dependence of strength has been much weaker than that observed in Fig. 3a. For hcp Mg single crystals with 90 and 100 nm diameters, the peak strength was 2.3 GPa^4^. For hcp Mg/Nb multilayers with 25 nm < *h* < 5 nm, the hardness remained around 2.48 GPa^14, 20^. Thus the outstanding 43% strength boost cannot be explained in terms of layer thickness alone. We postulate that the change in crystal structure from hcp to bcc leads to alterations in the intrinsic properties of the dislocations responsible for crystallographic slip, the types of slip systems that are activated, and the energetic barriers to move them. Additionally such changes will lead to differences in the atomic structure of the interface from a coherent bcc/bcc interface to an incoherent hcp/bcc. Interface response to deformation, particular in response to its interaction with dislocations, has been shown to be closely tied with its atomc structure^37^.

Further evidence of bcc Mg strengthening can be obtained from analyzing the plastic deformation involved from yield to peak strength. Figure 3b shows the strain hardening rates for the Mg/Nb 5 nm/5 nm and 50 nm/50 nm nanocomposites. The Mg/Nb 50 nm/50 nm nanocomposite exhibits a starting strain hardening rate of 35–40 GPa, which is about half its measured elastic modulus. The strain hardening rates for the Mg/Nb 5 nm/5 nm nanocomposite are even higher, around 50 GPa (~0.67*E*). These plastic strain hardening rate values are more than an order of magnitude higher than that those normally found in bulk hcp Mg, which is typically less than ~500 MPa. These results provide support that these dramatic increases in strength are a consequence of the bcc Mg phase.

Just as important as the outstanding strength is the two-fold improvement in the strain to failure (or strain to instability) for the bcc Mg composite (Fig. 3a and b). The 50 nm/50 nm Mg/Nb nanocomposite (where Mg is hcp) reaches a strain of 0.09 before softening, while the 5 nm/5 nm M g/Nb nanocomposite (where Mg is bcc) extends as much as 0.18 strain. This increase in strain to failure with further refinement in layer thickness is opposite of the trend conventionally seen in nanostructured metals. Typically for the same material system, the strain to failure decreases with decreases in grain size, crystal diameter or layer thickness^38, 39^. Further, the ability to strain harden over a large straining period implies higher ductility and formability for bcc Mg compared to hcp Mg.

More clues into the differences in plastic behavior of bcc versus hcp Mg can be gleaned from studying the pillar response under deformation. Figure 3c and d compare typical images of the bcc Mg/Nb and hcp Mg/Nb nanocomposite micropillars, respectively, at various strain levels (see also the *Supporting Information* section for movies of the micro-pillar deformations). The deformation behaviors of the two composites are strikingly different. For the hcp 50 nm/50 nm Mg/Nb nanocomposite (Fig. 3d), an instability occurs before the peak strength is reached followed by inhomogeneous deformation in multiple localized regions throughout the pillar. Other hcp nanomaterials respond in a similar manner when compressed along their c-axis, as is done here^29^. In contrast, the applied strain is accommodated more homogeneously in the bcc Mg/Nb nanocomposite. Other bcc nano structured metals, like steel, Ta, and Fe behave similarly in micro pillar compression, maintaining a high flow stress over an extended straining period^40^. However, a non-catastrophic deformation behavior is not necessary seen in all bcc nanostructured materials and is yet another desirable feature of this bcc Mg based material.

It is worth highlighting that the bcc Mg/Nb composites possess an unusually homogeneous response for a very finely nanostructured metal (~5 nm). Many factors could have contributed to its homogeneous deformation. First, bcc materials deform by at least 48 slip systems with relatively small differences in activation barrier compared to the fewer slip systems in hcp materials. DFT calculations of gamma surfaces have suggested that the same 48 slip systems common to bcc metals are favorable in bcc Mg^24^. Second, the interfaces permit co-deformation of the Mg and Nb nanolayers. Plastic deformation in the 5 nm layers occurs via discrete, single dislocations events. Individual dislocations nucleate from one boundary and get absorbed at another and do not pile up within the layers. Sustaining homogeneous plasticity relies on the transfer of dislocations across the interfaces to prevent pile-ups that localize deformation and generate stress concentrations. As shown in Fig. 1, the coherent bcc Mg/Nb has a cube-on-cube orientation relationship, which means that the same slip planes and slip directions have the same orientation on both sides of the interface. Under loading, the slip systems favored in one crystal are also favored in the other crystal; therefore, all slip transmission pathways are continuous, enabling co-deformation^41^. In this way, the coherent bcc Mg/Nb interface permits slip transfer, and thus homogeneous deformation, more so than the semi-coherent hcp Mg/Nb interface.

Here we show that nanostructured bcc Mg achieves a unique combination of ultra-high strength as well as an increased resistance to failure/instability. Grain refinement alone is not expected to lead to the same outstanding increases in strength and to any increases in ductility. Thus, the strength and ductility attained by the interface induced phase transformation to bcc Mg appears to be intrinsic.

These desirable properties of bcc Mg would need to be stable not only under mechanical strains, but also elevated temperatures. Many Mg alloy applications, such as for engine blocks, demand that the material withstand elevated temperatures without changes in strength and internal structure^42^. Both the hcp Mg and bcc Mg composites contain a high density of biphase interfaces, which, in prior studies of other material systems, have been shown to be more thermally stable^43, 44^ and resistant to slip transmission^45, 46^ and radiation tolerant^47^ than the grain boundaries in single phase constituents. To compare the thermal stability of bcc Mg relative to hcp Mg, the two composites were exposed to 373 K and 473 K (0.4*T*_*m*_ and 0.5*T*_*m*_, respectively, where *T*_*m*_ is the homologous temperature for Mg) for one hour. We compare their indentation modulus and hardnesses before and after exposure to these temperatures. Figure 4 shows the composite modulus and hardness before and after the high-temperature treatment. First we note that, before elevated temperature exposure, the hardness values of both materials are 2–3 times higher than the volume average of the nanocrystalline counterparts^33–35^, as discussed earlier. Second, and more interesting, the modulus and hardness of the 5 nm bcc Mg composites are just as thermally stable as those for the 50 nm hcp Mg composite. This is a remarkable result since finer nanolayered composites are usually less thermally stable than thicker ones, since the interconnecting grain boundaries along which atoms can diffuse are shorter^48, 49^. Here we see the opposite effect. The outstanding thermal stability of the bcc Mg is again thought to be a result of the coherent bcc Mg/Nb interface.

In summary, we show that bcc Mg/Nb nanolaminates are stronger and much more ductile than hcp Mg/Nb and exhibit thermal stability in hardness after exposure to temperatures of up to 0.5 times the homologous temperature of Mg. These outstanding properties are attributed to an interface-strain-induced transformation of Mg from hcp to bcc. The bcc Mg phase is a more ductile phase than hcp Mg and it makes a coherent interface with bcc Nb that is thermally stable. These findings can provide valuable insight into exploiting interface strain engineering to obtain more mechanically desirable, lightweight Mg.

## Materials and Methods

### Nanolayered composite fabrication

Both Mg and Nb layers were deposited in a magnetron sputtering system with a base pressure of 2 × 10^−8^ Torr. Both layers were deposited using DC magnetron sputtering at a process pressure of 3 millitorr with 300 watts of power on a 2-inch target. The deposition rates were 0.83 nm/sec for Mg and 0.22 nm/sec for Nb. The total film thicknesses for all samples were approximately 5 µm.

### Microscopy

Cross sectional TEM samples of the as-deposited films were prepared by mechanical polishing to a final thickness of 20–30 µm and a final finish of 1 µm with diamond lapping film, followed by ion-milling using a Gatan™ PIPS® instrument operating at 3–5 kV. The samples were examined using a Tecnai TF 30TM 300 kV TEM.

### Micropillar fabrication and testing

The micropillars were fabricated in a dual beam FEI Helios™ FIB SEM, using a beam of Ga + ions to remove the material and shape it in pillar form. All micro-pillars had height-to-width ratio of around 2:1 (5 µm:2.5 µm) and around six degrees of vertical taper. Due to the taper the diameter measured at the pillar top (the smallest measurement) was used for stress calculations. 4–5 pillars of each layer thickness were tested in micro-compression. *In-situ* uniaxial compression tests were conducted to capture the local microstructural evolution in the course of deformation.

The *in-situ* testing was conducted in a nanomechanical instrument, which is comprised of a nano-mechanical tester (Hysitron PI-85™) inside of a SEM (FEI Helios™). The pillars were compressed with a flat punch conductive diamond tip of 20 µm diameter, at a nominal displacement rate of 2 nm s^−1^. The continuously captured image scans were recorded as a video file during the test.

### Compliance correction and stress-strain calculations

Strain measurements during micropillar compression testing is often ambiguous due to two main factors: (a) misalignment of the indenter tip with respect to the pillar, which makes it difficult to locate the point of full contact during loading between the indenter and the pillar and (b) compliance of the substrate on which the pillar is standing. In order to correct the strain measurements during micro-pillar compression, we followed a 2-step approach as described below.

Firstly, we choose the unloading segments of the tests that were unloaded at low strain levels (*ϵ* ~ 0.1), i.e., after full contact has been established for composite modulus measurements. Assuming the unloading to be elastic, we can write ${h}_{total}={h}_{pillar}+{h}_{substrate}$., where the total displacement (${h}_{total}$) includes the combined effects of the displacement of the pillar (${h}_{pillar}$) as well as the (elastic) displacement of the substrate (${h}_{substrate}$). In order to calculate the correct strain on the pillar, this additional compliance of the substrate needs to be subtracted.

${h}_{substrate}$ was measured from image scans recorded during the unloading segment of the *in-situ* SEM tests. The composite modulus (as then calculated from corrected displacement of the pillar (${h}_{pillar}$), the measured load and the pillar dimensions. 3–4 micropillar compression tests were used to calculate the average ± standard deviation of the measured modulus values.

In the second step we use the ${E}_{corrected}$ values to correct for the calculate the actual strain (${\varepsilon }_{corrected}$) on the micro-pillar as follows:

$${\varepsilon }_{corrected}={\varepsilon }_{uncorrected}-\frac{\sigma }{{E}_{uncorrected}}+\frac{\sigma }{{E}_{corrected}},$$

where ${E}_{uncorrected}$ is the initial (uncorrected) slope of the elastic loading section, ${\varepsilon }_{uncorrected}$ is the original strain (before correction), and $\sigma$ is the measured stress. The corrected stress-strain plot can now be used to accurately measure the yield strain, strain hardening etc.

A small amount of non-linearity can be observed at very low strains in Fig. 3a. This is due to initial misalignment of the micropillar and the indenter flat punch at the beginning of the test. This non-linearity was taken into account when computing the 0.2% offset yield stress values. Thus, the 0.2% offset was computed as an offset from the linear portion of elastic segment *after* the very initial non-linearity.

### Nanoindentation hardness

The hardness measurements were conducted using a diamond Berkovich tip using two nanoindentation machines - the Hysitron Triboindenter™ and the Agilent XP™. 50 tests per sample were made in each nanoindenter machine using a 10 sec (loading) – 100 sec (hold) – 10 sec (unloading) cycle. The longer hold time of 100 sec was chosen to offset any potential creep effects after the high temperature exposures. Tests were done to a maximum displacement of 200 nm for the Hysitron Triboindenter™ (instrument limit) and up to a maximum displacement of 200 and 400 nm for the Agilent XP™. These choices of indentation depth ensured that in each case the indenter was sampling regions within and beyond at least one bilayer thickness (2λ).

### Thermal stability tests

For the thermal stability measurements, the 5 nm/5 nm and 50 nm/50 nm Mg/Nb nanocomposites were heated to annealing temperatures of 100 °C (0.4*T*_*m*_, where *T*_*m*_ is the homologous temperature for Mg) and 200 °C (0.5*T*_*m*_) for one hour and then allowed to cool down to room temperature. Hardness measurements were conducted before and after the thermal treatments.

## Electronic supplementary material

Supporting Information (SI)

Video S1

Video S2
