# Supplementary material for: Strong, Ductile, and Thermally Stable bcc-Mg Nanolaminates
Source: Sci Rep. 2017 Aug 15;7:8264. doi: 10.1038/s41598-017-08302-5 (PMC5557767; doi:10.1038/s41598-017-08302-5)
Supplement: Supplementary file 1 — Supporting Information (SI) [file 41598_2017_8302_MOESM1_ESM.pdf]

Strong, Ductile, and Thermally Stable bcc-Mg Nanolaminates

Siddhartha Pathak [1*], Nenad Velisavljevic [2], J. Kevin Baldwin [3], Manish Jain [1], Shijian Zheng [3,4], Nathan A. Mara [3,5], Irene J. Beyerlein [6]

[1] Chemical and Materials Engineering, University of Nevada, Reno, NV
[2] Shock and Detonation Physics, Los Alamos National Laboratory, Los Alamos, NM
[3] Center for Integrated Nanotechnologies, Los Alamos National Laboratory, Los Alamos, NM
[4] Shenyang National Laboratory for Materials Science, Institute of Metal Research, Chinese Academy of Sciences, Shenyang 110016, China
[5] Institute for Materials Science, Los Alamos National Laboratory, Los Alamos, NM
[6] Mechanical Engineering Department, Materials Department, University of California at Santa Barbara, Santa Barbara, CA 93106, USA

*corresponding author: spathak@unr.edu, (775) 784-7098, 1664 N Virginia St, Mail Stop 0388, University of Nevada, Reno, NV 89557-0388 USA

Supporting Information (SI) available

Videos S1 and S2 illustrate the *in-situ* deformation of Mg/Nb 5nm/5nm and Mg/Nb 50nm/50nm nanocomposites respectively when compressed at a 2 nm/s displacement rate (~4×10^{-4} strain rate). The online video files, synchronized with its engineering stress-strain response, are shown at 48 and 37 times their original speeds respectively.
